# Supplementary material for: Insightful classification of crystal structures using deep learning
Source: Nat Commun. 2018 Jul 17;9:2775. doi: 10.1038/s41467-018-05169-6 (PMC6050314; doi:10.1038/s41467-018-05169-6)
Supplement: Supplementary file 1 — Supplementary Information [file 41467_2018_5169_MOESM1_ESM.pdf]

Supplementary Information for:

Insightful classification of crystal structures using deep learning

1 Supplementary Note 1

Here, we show a complete comparison between a state-of-the-art method (Spglib) and the proposed approach in classifying defective structures when random displacements are applied (Supplementary Table 1), or vacancies are created (Supplementary Table 2), as described in the main text. The thresholds indicated as "tight", "medium", "loose" in the tables below (and in Table 1 in the main text) are obtained with the following choices of Spglib parameters: "tight"= {`symprec`= 1×10^{-3}Å, `angle_tolerance`= 1° }; "medium"= {`symprec`= 1×10^{-2}Å, `angle_tolerance`= 1° }; "loose"={`symprec`= 1×10^{-1}Å, `angle_tolerance`= 5° }. Finally, concerning defective structures obtained by chemical substitution, we find that our method is always able to identify the correct crystal class.

	Random Displacements (σ)										
	0.001Å	0.002Å	0.003Å	0.004Å	0.005Å	0.01Å	0.02Å	0.04Å	0.06Å	0.08Å	0.10Å
Spglib (tight)	0.00	0.00	0.00	0.00	0.00	0.00	0.00	0.00	0.00	0.00	0.00
Spglib (medium)	73.70	0.00	0.00	0.00	0.00	0.00	0.00	0.00	0.00	0.00	0.00
Spglib (loose)	99.99	99.99	99.99	99.99	99.99	75.22	0.00	0.00	0.00	0.00	0.00
**This work**	100.00	100.0	100.00	100.00	100.00	100.00	100.00	100.00	100.00	99.97	99.91

Supplementary Table 1. Accuracy in identifying the correct (most similar) crystal class in the presence of defects. The defective structures are calculated randomly displacing atoms according to Gaussian distribution with standard deviation σ. The accuracy values are in percentage.

	Vacancies (η)											
	1 %	2 %	5 %	10 %	15 %	20 %	25 %	30 %	40 %	50 %	60 %	70 %
Spglib (tight)	0.02	0.00	0.00	0.00	0.00	0.00	0.00	0.00	0.00	0.00	0.00	0.00
Spglib (medium)	0.02	0.00	0.00	0.00	0.00	0.00	0.00	0.00	0.00	0.00	0.00	0.00
Spglib (loose)	0.01	0.00	0.00	0.00	0.00	0.00	0.00	0.00	0.00	0.00	0.00	0.00
**This work**	100.00	100.00	100.00	100.00	100.00	100.00	100.00	100.00	100.00	99.56	97.18	89.05

Supplementary Table 2. Accuracy in identifying the correct (most similar) crystal class in the presence of defects. The defective structures are calculated randomly removing η% of the atoms, thus creating vacancies. The accuracy values are in percentage.

2 Supplementary Note 2

Since in the main text we have shown that the diffraction descriptor is well-behaved with respect to defects (Fig. 2), and that the neural network can extrapolate correctly to intermediate structures (see Fig. 4a), let us now address the question if our method can distinguish ordered structures with defects and amorphous structures. There is an empirical (universal) criterion - the so-called Lindemann melting rule[1] - to predict the solid-liquid transition: it states that this occurs when the ratio between the root-mean-square fluctuations about lattice positions and the nearest neighbor distance (termed Lindemann parameter) exceeds a certain threshold. Here, we investigate whether structures, to which random displacements to the atomic positions are applied, are recognized as amorphous structures when the resulting Lindemann parameter exceeds the threshold. In practice, we generate structures with Lindemann parameter ranging from 0 (pristine) to 0.3 (amorphous) from a subset of fcc, bcc, and bct_{139} structures belonging to the pristine dataset. In Supplementary Fig. 1 we plot the classification probability of all classes versus Lindemann parameter. Let us here discuss in detail the case of fcc shown in Supplementary Fig. 1a. Up to a Lindemann parameter of 0.075, the model classifies these disordered structures as fcc with probability close to 1, and zero standard deviation. In the range 0.1-0.2, however, the fcc probability classification drops significantly, just as the standard deviation increases: the neural network is now less confident on its predictions. For Lindemann parameters greater than 0.2 the structures are amorphous, and therefore the neural network classification is no longer meaningful: this is reflected in the very large standard deviation of the classification probability distributions. It is interesting to note that the drop of confidence in the neural network predictions falls in the range of commonly accepted critical values for the Lindemann parameter (0.1-0.2)[2].

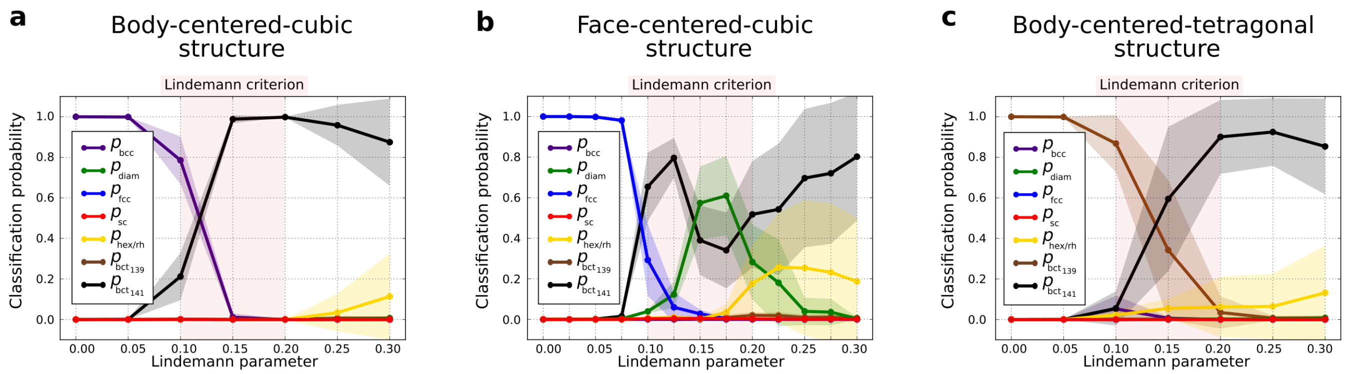

Supplementary Figure 1. From ordered to amorphous structures: the case of (a) fcc, (b) bcc, and (c) bct_{139} crystal structures. The distributions of classification probability of all crystal classes are shown in each plot. The drop in the classification probability distribution for the fcc (a), bcc (b), and bct_{139} (c) occurs around to the range of commonly accepted Lindemann parameters (shaded pink area between 0.1 and 0.2) for a solid to liquid transition (see text for more details).

This is interesting, especially given that our model was built using exclusively pristine structures, and has thus no information regarding disordered structures. The situation for bcc and bct_{139} classes (shown in Supplementary Fig. 1b and 1c, respectively) follow a quantitative different but qualitatively comparable trend.

3 Supplementary Note 3

The raw data used to generate the pristine dataset (corresponding to the AFLOWLIB elemental solid database) can be downloaded from NOMAD Repository with the following links:

1. http://data.nomad-coe.eu/raw-data/data/Rnh/Rnh_4DFTJQgTSOib4e4d-5GByiTVB.zip
2. http://data.nomad-coe.eu/raw-data/data/R10/R10ncY1AZG6X9y-Nj8F0_DiN8NeLD.zip
3. http://data.nomad-coe.eu/raw-data/data/RsL/RsLoZhSAdK0BopfI2T4B5pLfMyjVN.zip
4. http://data.nomad-coe.eu/raw-data/data/RMG/RMGpPc3B_HiR0D-oLE4ND66HmYdH-.zip
5. http://data.nomad-coe.eu/raw-data/data/Re2/Re2mnhOAs6ZNqvTY1p-W2RavinjOM.zip
6. http://data.nomad-coe.eu/raw-data/data/R9u/R9usAWjw2xq9F8zW-66jyCyeDLlDa.zip
7. http://data.nomad-coe.eu/raw-data/data/Rkx/RkxmUCgPxt-9xDdIpr5xqPQK8PC9H.zip
8. http://data.nomad-coe.eu/raw-data/data/Rdz/RdzeezGR0W5wGEpGYEqOq7AygYS9J.zip
9. http://data.nomad-coe.eu/raw-data/data/Rc_/Rc_XxYadb0ZlfBVLqCNo-EtVocxv8.zip
10. http://data.nomad-coe.eu/raw-data/data/RKX/RKXqE9xPCiLlufNK0n4pbtzdbID5H.zip
11. http://data.nomad-coe.eu/raw-data/data/Ryv/RyvdvBLf1QdM5QJ_8DVve7CknkdK5.zip
12. http://data.nomad-coe.eu/raw-data/data/Rut/Rut3qcReY6SJO6fIJ5jangTSlMjaQ.zip
13. http://data.nomad-coe.eu/raw-data/data/Reg/Reg0D-KojGnrw51EYl2Q2rCYOIfJM.zip
14. http://data.nomad-coe.eu/raw-data/data/RSk/RSkoltrNkpZwp1xpi_Oj1jO4IndC5.zip
15. http://data.nomad-coe.eu/raw-data/data/RA-/RA-tqhSlH5idfPW_3UxE80I7BBL6s.zip
16. http://data.nomad-coe.eu/raw-data/data/Ray/RayT1o-XjyZaWdlVS_Fk8nssdO1w9.zip
17. http://data.nomad-coe.eu/raw-data/data/Rlb/RLbbgx7klbZ7ZdO5O_YABQGjBOZ9g.zip
18. http://data.nomad-coe.eu/raw-data/data/RcC/RcC8TDWGWCtQLhWeB2a1N8y9Q7y4r.zip
19. http://data.nomad-coe.eu/raw-data/data/Ra8/Ra8nAuJOgxGwSytw1scU5BTeB3ozo.zip

Additional data including atomic structures and two-dimensional diffraction fingerprints of the pristine dataset are available from the Harvard Dataverse: https://doi.org/10.7910/DVN/ZDKBRF.

Supplementary References

1. F. A. Lindemann, Physik. Z. **11**, 609 (1910).

2. D. A. Young, Phase Diagrams of the Elements, University of California Press (1991).
