# Supplementary material for: Insightful classification of crystal structures using deep learning
Source: Nat Commun. 2018 Jul 17;9:2775. doi: 10.1038/s41467-018-05169-6 (PMC6050314; doi:10.1038/s41467-018-05169-6)
Supplement: Supplementary file 2 — Peer Review Report [file 41467_2018_5169_MOESM2_ESM.pdf]

Reviewers' comments:

Reviewer #1 (Remarks to the Author):

The present manuscript proposes deep learning approach to automatically classify structures by crystal symmetry. The way to represent crystal system as an image and classify them using deep architecture as proposed is, however, not new (Please see IUCrJ recent issues). The scope of the present work is very limited, because it deals with the classification of a cubic system to only four space groups (Fm-3m, Fd-3m, Im-3m, and Pm-3m). The effort used to unravel the neural network internal operations in terms of attentive response maps is however, appreciable. In my opinion nature communication is not a good platform for this kind of study and therefore, the article should better be submitted to a journal close to its application domain. The usability of deep learning in the field of crystallography should be rated by crystallographers. I recommend the authors to submit this manuscript to either IUCrJ or Journal of applied crystallography. The existing rule-based academia generally has a negative impression of deep learning that might switch their routines. To overcome such skepticism, Google DeepMind also declared this year that they will focus on scientific issues by stopping the entertaining themes. This means that the deep learning is no longer a sort of just a broadly interesting new trend but a substantial technology that has to emulate with existing scientific routines and eventually defeat them. Neither Nature nor IEEE journals would be good media for this paper in this context. Although this manuscript could be accepted in crystallography journals, the authors should clearly describe that this paper at the current status seems far from complete in comparison to the conventional x-ray diffraction techniques. Some of recent success stories achieved by the deep learning were evaluated of high standard not because they were a novel approach but because they defeated existing routines, e.g., the accuracy of the deep learning-based picture classification was better than any other rule-based approaches and DQN plays Atari games better than humans. Since such classification for four cubic space groups is an extremely simple routine in the field, no need to adopt the deep learning to deal with such simple issue.

Some other issues which need to be addressed in the manuscript are:

The term "crystals" used at many places in the manuscript, appears to be awkward and confusing with mineralogy rather than crystallography, for example "---introduce an automatic procedure to classify crystals...", "--- we introduce a new way to represent crystals---" , etc. It should be rather mentioned as classification of crystal symmetry or anything relevant to crystallography.

How does the authors ensure that in creation of defect structure for data set by random (25%, 50% and 75%) substitution with other atoms does not lead to any change and overall cubic symmetry? It doesn’t make any sense because such substitutions would definitely make a change in lattice parameters and in turn induce a phase transition or some different ordering.

Classification of a cubic system is restricted to only four space groups such as Fm-3m, Fd-3m, Im-3m, and Pm-3m using deep learning, which could be very easily done using x-ray diffraction techniques on the basis of systematic absence. How to deal with a defect structure which do not fall under these four space groups?

Reviewer #2 (Remarks to the Author):

This work describes the use of a deep learning framework to classify crystal structures. First, the two-dimensional diffraction pattern fingerprints are generated for each crystal structure, and this representation forms the descriptor for the structure. Image recognition deep learning networks (specifically the convolutional neural network) are then trained on a set of cubic crystal structures (FCC, BCC, diamond, and simple cubic) extracted from the AFLOWLIB online database. The model

is then tested by shifting or removing atoms at random from the structures, and the authors show that the model can still successfully identify the original parent structure.

This work is a novel approach to the task of crystal structure identification, which is a very important problem in computational material science. Knowledge of the crystal structure is vital for reducing the size of the cells that need to be calculated, and for identifying the appropriate paths in reciprocal space to be used for calculating the electronic band structure. This approach has the advantage of being very robust, and can determine the underlying symmetry of highly disordered systems without the need for user-defined tolerances, which is particularly important for high-throughput analysis of large data sets. It can also identify structures that are intermediate between two different crystal lattices as being such. Finally, this approach can identify the transition from ordered to amorphous structures, putting the transition in the same value range for the Lindemann parameter as that commonly accepted for this transition.

The novelty and advantages offered by this approach make this work suitable for publication in Nature Communications. However, the authors should consider the following recommended changes, which should improve the quality and impact of the work:

1. The main weakness of this work is that only cubic symmetry materials are investigated. If possible, including other structures, and demonstrating that the method can correctly identify the transitions from cubic to tetragonal to orthorhombic, etc., would increase the impact of this work. Comparisons to conventional symmetry analysis packages, such as FINDSYM or spglib, would also be useful, particularly details such as the differences in computational cost for analyzing structures, or comparisons of the robustness of the different approaches. This would put the strengths of the approach described here into perspective. The relevant citations for these two packages should be included in the article, to properly reflect the current state of the art in crystal structure analysis.

2. In addition, more details should be given on how the initial training set was selected and characterized. Are these materials based on structures taken from experimental databases such as the ICSD, or are they hypothetical structures generated by decorating prototypes? Were these initially selected based on the symmetry labels provided by the AFLOWLIB database? Were additional checks on the symmetry performed by the authors? If so, then details such as the tolerances used should be provided to give an accurate picture of how the training set was constructed. If the AFLOWLIB REST-API or AFLOW code were used to retrieve or characterize these structures, then the relevant papers should also be cited.

3. Finally, although the paper is generally well-written, there are some typos and grammatical errors that should be corrected prior to publication. These include “loose” instead of “lose” in the introduction, “raise” instead of “rise” in first subsection in the results, using “such” when “this” would be more correct (e.g. “Such descriptor compactly encodes” should be “This descriptor compactly encodes”). In the Discussion section, “possess” should be “possesses”, “crystals” should be “crystal” and “required” should be “require”.

Reviewer #3 (Remarks to the Author):

The ability to determine crystal structures from diffraction data is of such great importance that it's difficult to overstate. Twenty-one Nobel prizes have been awarded for such work, the most recent being in 2016. The artificial-intelligence community now promise to turn the field on its head extracting crystal-structure information directly from diffraction patterns without directly tackling the inverse problem of phase extraction. The present manuscript accomplishes this via a deep-learning technique based on a convolutional neural network.

This manuscript has notable similarities to another recent paper by Park et al. [IUCrJ 4, 486-494, 2017], though it was not cited, possibly because it was published only a month ago. Because both papers employ convolutional neural networks in order to classify crystal-structure information from diffraction data, it is worth briefly comparing the two.

Park's training data was experimental powder-diffraction patterns from a wide variety of materials, obtained from a 3rd-party database. Their scope was broad and synthetic-chemistry relevant, though their classification was fairly course (only crystal symmetry info).

The present manuscript's training data was simulated 110-oriented single-crystal diffraction images from displacively and occupationally defective computer models from a handful of fairly simple cubic materials. Their scope was narrow and materials-simulation relevant, though the classification was very fine (actual crystal structure).

Thus, judged by scope, audience, and classification type, the two papers are very different.

I am not a machine-learning expert and so can't comment on the novelty or importance of the specific machine-learning approach used by the authors. But I do wish that the present abstract were more clear about the training scope and classification type. My only meany criticism of the paper is that the scope so narrow (110-oriented images from un-complicated cubic crystals) as to prove only the concept rather than demonstrating a real materials breakthrough. But I feel that idea presented has great potential. Because crystal defects exist in such variety, and have such a detrimental affect on traditional structure-determination methods, this aspect of the present manuscript is particularly interesting.

Manuscript: "The face of crystals: insightful classification using deep learning"
Reply to the Reviewers' comments

Reviewer #1 (Remarks to the Author):

The present manuscript proposes deep learning approach to automatically classify structures by crystal symmetry. The way to represent crystal system as an image and classify them using deep architecture as proposed is, however, not new (Please see IUCrJ recent issues). The scope of the present work is very limited, because it deals with the classification of a cubic system to only four space groups (Fm-3m, Fd-3m, Im-3m, and Pm-3m). The effort used to unravel the neural network internal operations in terms of attentive response maps is however, appreciable.
In my opinion nature communication is not a good platform for this kind of study and therefore, the article should better be submitted to a journal close to its application domain. The usability of deep learning in the field of crystallography should be rated by crystallographers. I recommend the authors to submit this manuscript to either IUCrJ or Journal of applied crystallography.

1.1) We assume that the article the referee is referring to is Park *et al.* [IUCrJ 4, 486-494, 2017, Ref.17 in the revised version of the manuscript]; if this is the case, then the sentence *"The way to represent crystal system as an image and classify them using deep architecture as proposed is, however, not new"* is both simplistic, and inaccurate.
In Ref. 17, Park *et al*. use as input powder X-ray diffraction (XRD) data calculated from entries in the ICSD database. These XRD data are 1D data, since they describe the intensity I as a function of the scattering angle, I(2θ). It is however well-known that small changes in the lattice parameters can cause large changes in the scattering angle and therefore in the powder XRD pattern, as also pointed out by the authors of Ref. 17 themselves (just below Eq. 1 in Ref. 17). This could explain the poor performance of their model, and its inability to generalize to novel structures, despite the very large neural network employed (~10^6-10^7 parameters). Moreover, we expect this to be very problematic for also defective structures, which however are not considered in Ref. 17.
In our work, we propose a robust and compact representation to capture average long-range order in crystal structures. Being an image, it is a 2D object (and not 1D as in Ref.17). To the best of our knowledge, this is the first time that an accurate, automatic classification of heavily defective crystal structures is accomplished.

1.2) The applicability domain of our work is most certainly not limited to crystallographers, but encompasses both the computational and experimental materials science communities (e.g. high-throughput computations and atom probe tomography experiments), but also researchers at the intersection between applied machine learning and materials science.

1.3) We have generalized our method in order to describe systems beyond cubic symmetry. The crystal structures considered in the revised version of the manuscript accounts for more than 80% of the structures adapted by elemental solids under standard conditions. Please see the revised version of the manuscript, and point 1.6) below.

The existing rule-based academia generally has a negative impression of deep learning that might switch their routines. To overcome such skepticism, Google DeepMind also declared this year that they will focus on scientific issues by stopping the entertaining themes. This means that the deep learning is no longer a sort of just a broadly interesting new trend but a substantial technology that has to emulate

with existing scientific routines and eventually defeat them. Neither Nature nor IEEE journals would be good media for this paper in this context. Although this manuscript could be accepted in crystallography journals, the authors should clearly describe that this paper at the current status seems far from complete in comparison to the conventional x-ray diffraction techniques.
Some of recent success stories achieved by the deep learning were evaluated of high standard not because they were a novel approach but because they defeated existing routines, e.g., the accuracy of the deep learning-based picture classification was better than any other rule-based approaches and DQN plays Atari games better than humans.

1.4) We definitely agree with the referee's statement "the deep learning […] has to emulate with existing scientific routines and eventually defeat them."
As it is clear from Table 1, S1 and S2, we have accomplished exactly this: defeat conventional techniques from obtaining the most similar crystal-structure prototype for defective systems. Our work is most certainly not "*an entertaining theme*", but solves a relevant scientific issue, as also acknowledged by Reviewer 2 and Reviewer 3.

Since such classification for four cubic space groups is an extremely simple routine in the field, no need to adopt the deep learning to deal with such simple issue.
1.5) We point out that our goal is not to classify experimental x-ray diffraction images, but to automatically classify – possible noisy and incomplete – three-dimensional structural data, for which no experimental x-ray measurements are present nor possible.
To clarify this even further, we have added the following paragraph to the revised version of the manuscript:
"Here, we propose a novel procedure to efficiently represent and classify potentially noisy and incomplete three-dimensional materials science structural data according to their crystal symmetry (and not to classify x-ray diffraction images, or powder x-ray diffraction data[17]). These three-dimensional structural data could be for example atomic structures from computational materials science databases, or elemental mappings from atom-probe tomography experiments."
Tables 1, S1 and S2 present a comparison between a current state-of-the-art method (Spglib) for crystal structure analysis and our proposed approach. The results clearly demonstrate that automatic crystal-structure classification of defected structures is anything but "*extremely simple routine in the field*", and that our method is far superior with respect to the current state-of-the-art for defective systems.

1.6) Regarding the limitation of cubic systems, in the revised manuscript we present a generalization of our method to non-cubic structures. Our dataset now comprises structures belonging to the following space groups: 139 (body-centered-tetragonal), 141 (body-centered-tetragonal), 166 (rhombohedral), 194 (hexagonal), 221 (simple cubic), 225 (face-centered-cubic), 227 (diamond), and 229 (body-centered-cubic). The scientific relevance of this dataset is proved by the fact that more than 80% of elemental solids under standard conditions adopt one of the crystal structures considered in this work.

Some other issues which need to be addressed in the manuscript are:

The term "crystals" used at many places in the manuscript, appears to be awkward and confusing with mineralogy rather than crystallography, for example "---introduce an automatic procedure to classify crystals...", "--- we introduce a new way to represent crystals---" , etc. It should be rather mentioned as classification of crystal symmetry or anything relevant to crystallography.

1.7) We do not see how the term "crystals" could confuse the reader, given that our article is clearly on

materials science, and has nothing to do with mineralogy. This was already stated repeatedly in the abstract and in the manuscript itself. Just a few examples: in the abstract, "*A reliable identification of lattice symmetry*"; "*Here, we propose a new machine-learning-based approach to automatically classify structures by crystal symmetry*". Already in the first sentence of the manuscript, the scope of the article is clearly defined: "*Crystals play a crucial role in material science*".
However, since we want to be absolutely sure that every reader of Nature Communications clearly understand the scope of the paper, we have followed the referee's suggestions and made the appropriate changes in the manuscript.

How does the authors ensure that in creation of defect structure for data set by random (25%, 50% and 75%) substitution with other atoms does not lead to any change and overall cubic symmetry? It doesn't make any sense because such substitutions would definitely make a change in lattice parameters and in turn induce a phase transition or some different ordering.

1.8) The defective structures considered in our work are obtained from the pristine (parental) one, without optimizing the structures. In fact, we are only interested in testing if our approach can successfully identify the original parent crystal structure. The same reasoning applies to random displacements and vacancies.

Classification of a cubic system is restricted to only four space groups such as Fm-3m, Fd-3m, Im-3m, and Pm-3m using deep learning, which could be very easily done using x-ray diffraction techniques on the basis of systematic absence. How to deal with a defect structure which do not fall under these four space groups?

1.9) This is true if one wants to classify experiment x-ray diffraction data. However, as discussed in detail at point 1.5), our purpose is to automatically classify three-dimensional structural data (without performing x-ray experiments). As also stated above (Point 1.6), a generalized version of our method beyond the cubic crystal systems is presented in the revised version of the manuscript.

Reviewer #2 (Remarks to the Author):

This work describes the use of a deep learning framework to classify crystal structures. First, the two-dimensional diffraction pattern fingerprints are generated for each crystal structure, and this representation forms the descriptor for the structure. Image recognition deep learning networks (specifically the convolutional neural network) are then trained on a set of cubic crystal structures (FCC, BCC, diamond, and simple cubic) extracted from the AFLOWLIB online database. The model is then tested by shifting or removing atoms at random from the structures, and the authors show that the model can still successfully identify the original parent structure.

This work is a novel approach to the task of crystal structure identification, which is a very important problem in computational material science. Knowledge of the crystal structure is vital for reducing the size of the cells that need to be calculated, and for identifying the appropriate paths in reciprocal space to be used for calculating the electronic band structure. This approach has the advantage of being very robust, and can determine the underlying symmetry of highly disordered systems without the need for user-defined tolerances, which is particularly important for high-throughput analysis of large data sets. It can also identify structures that are intermediate between two different crystal lattices as being such. Finally, this approach can identify the transition from ordered to amorphous structures, putting the

transition in the same value range for the Lindemann parameter as that commonly accepted for this transition.

The novelty and advantages offered by this approach make this work suitable for publication in Nature Communications. However, the authors should consider the following recommended changes, which should improve the quality and impact of the work:
1. The main weakness of this work is that only cubic symmetry materials are investigated. If possible, including other structures, and demonstrating that the method can correctly identify the transitions from cubic to tetragonal to orthorhombic, etc., would increase the impact of this work. Comparisons to conventional symmetry analysis packages, such as FINDSYM or spglib, would also be useful, particularly details such as the differences in computational cost for analyzing structures, or comparisons of the robustness of the different approaches. This would put the strengths of the approach described here into perspective. The relevant citations for these two packages should be included in the article, to properly reflect the current state of the art in crystal structure analysis.

We thank the referee for his/her constructive criticisms. Following the referee useful suggestions, we have applied the following modifications.

2.1) We have generalized our procedure to systems beyond the cubic symmetry. We now include the following space groups: 139 (body-centered-tetragonal), 141 (body-centered-tetragonal), 166 (rhombohedral), 194 (hexagonal), 221 (simple cubic), 225 (face-centered-cubic), 227 (diamond), and 229 (body-centered-cubic). These are all systems belonging to centrosymmetric space groups represented with more than 50 calculations in the AFLOWLIB elemental solids database. This dataset covers the large majority (more than 80%) of the crystal structures adopted by elemental solids under standard conditions.

2.2) We have included a detailed comparison with the symmetry package Spglib using three different thresholds setting (tight, medium, loose) in Table 1, S1 and S2. We have also added the citation to the Spglib website (https://atztogo.github.io/spglib/) since we could not find any publications on the Spglib code itself (and we had already included the citations to the “Specific algorithms” listed in the Spglib web page in the previous version of our manuscript).
To reflect the current state-of-the-art in crystal structure analysis, we have also included the following sentence in the introduction:
“*this is implemented in existing symmetry packages such as FINDSYM[7], Platon[8], Spglib[9-11], and most recently the self-consistent, threshold-adaptive AFLOW-SYM[12].*”

2.3) As suggested by the referee, in the revised version of the manuscript we show that our approach correctly identifies structural transitions also beyond the cubic crystal system. In particular, we show that we are able to correctly identify the following structural transitions: i) body-centered-cubic to rhombohedral, ii) rhombohedral to simple-centered-cubic iii) rhombohedral to face-centered-cubic.
These new results are presented in Fig. 4b of the revised version of the manuscript, and described in subsection “The model performance”, from: “*We conclude our model exploration applying our classification procedure on a structural transition path …*” till “*as being rhombohedral for all other values of q.*”
We underline that, as in the previous example within the cubic crystal system, the training of the model os performed solely on pristine structures. The combination of the physical/geometrical aspects captured by the the descriptor and the smoothness of the metric induced by the model allows for a error-free identification of the transitions among crystal strucutres, without any training with intermediate structures.

2. In addition, more details should be given on how the initial training set was selected and characterized. Are these materials based on structures taken from experimental databases such as the ICSD, or are they hypothetical structures generated by decorating prototypes? Were these initially selected based on the symmetry labels provided by the AFLOWLIB database? Were additional checks on the symmetry performed by the authors? If so, then details such as the tolerances used should be provided to give an accurate picture of how the training set was constructed. If the AFLOWLIB REST-API or AFLOW code were used to retrieve or characterize these structures, then the relevant papers should also be cited.

2.4) The construction of the training set was described in subsection "Dataset" of the section "Method". Quoting the earlier version of the manuscript: "*Our pristine dataset consists of materials from the AFLOWLIB elemental solid database […] as determined by a symmetry-based approach*."
However, we certainly agree with the referee that it is useful to provide additional details regarding the training set construction. Therefore, we have implemented the following changes.

a) We explicitly mention in the text the use of AFLOWLIB calculated data and Spglib in the training set construction:
"For every calculation in the AFLOWLIB elemental solid database[49,50], we determine its space group using a symmetry-based approach as implemented by the Spglib code".
The details regarding the exact Spglib tolerance used for the training set construction are included in the subsection "Dataset" of the section "Method":
"*The space group is determined using the Python Materials Genomics (pymatgen)[62] wrapper around the Spglib[11] library with* `symprec`*=10^{-3} Å and* `angle_tolerance`*=1°"*

b) Previously, we did not include the citation to the AFLOWLIB REST-API because we retrieved the data from the NOMAD Archive. However, now that we are using the AFLOW code to generate the prototypes used in the structural transition presented in Fig. 4b, we now include the citations to the AFLOWLIB REST-API (Taylor 2014, Ref. 50) and to the AFLOW Library of Crystallographic Prototypes (Mehl 2016, Ref. 51). The AFLOW code was already cited in the previous version of the manuscript.

3. Finally, although the paper is generally well-written, there are some typos and grammatical errors that should be corrected prior to publication. These include "loose" instead of "lose" in the introduction, "raise" instead of "rise" in first subsection in the results, using "such" when "this" would be more correct (e.g. "Such descriptor compactly encodes" should be "This descriptor compactly encodes"). In the Discussion section, "possess" should be "possesses", "crystals" should be "crystal" and "required" should be "require".

2.5) We thank the referee for pointing this out. His/her suggested corrections have been implemented in the current version of the manuscript.

Reviewer #3 (Remarks to the Author):

The ability to determine crystal structures from diffraction data is of such great importance that it's difficult to overstate. Twenty-one Nobel prizes have been awarded for such work, the most recent being in 2016. The artificial-intelligence community now promise to turn the field on its head extracting crystal-structure information directly from diffraction patterns without directly tackling the inverse

problem of phase extraction. The present manuscript accomplishes this via a deep-learning technique based on a convolutional neural network.

This manuscript has notable similarities to another recent paper by Park et al. [IUCrJ 4, 486-494, 2017], though it was not cited, possibly because it was published only a month ago. Because both papers employ convolutional neural networks in order to classify crystal-structure information from diffraction data, it is worth briefly comparing the two.

Park’s training data was experimental powder-diffraction patterns from a wide variety of materials, obtained from a 3rd-party database. Their scope was broad and synthetic-chemistry relevant, though their classification was fairly course (only crystal symmetry info).

The present manuscript's training data was simulated 110-oriented single-crystal diffraction images from displacively and occupationally defective computer models from a handful of fairly simple cubic materials. Their scope was narrow and materials-simulation relevant, though the classification was very fine (actual crystal structure).

Thus, judged by scope, audience, and classification type, the two papers are very different.

I am not a machine-learning expert and so can't comment on the novelty or importance of the specific machine-learning approach used by the authors. But I do wish that the present abstract were more clear about the training scope and classification type. My only meany criticism of the paper is that the scope so narrow (110-oriented images from un-complicated cubic crystals) as to prove only the concept rather than demonstrating a real materials breakthrough. But I feel that idea presented has great potential. Because crystal defects exist in such variety, and have such a detrimental affect on traditional structure-determination methods, this aspect of the present manuscript is particularly interesting.

3.1) As pointed out by the referee, the relation between our work and Park et al. [IUCrJ 4, 486-494, 2017, Ref.17 in the revised version of the manuscript] is indeed only superficial, the only similarities being the use of convolutional neural networks (albeit 1D for Park *et al.*, and 2D for our work) to classify crystal structures. Nevertheless, we would like to clarify this matter further in order to put our work into perspective.
We feel that the referee statement:
“[...] both papers employ convolutional neural networks in order to classify crystal-structure information from diffraction data, [...]”
could be somewhat misleading.
If it is indeed true that both works use diffraction data to classify crystal structures, the type and origin of these “diffraction data” are very different. Park *et al*. represent crystal structures using powder X-ray diffraction (XRD) patterns while we represent crystal structures by our two-dimensional diffraction fingerprint (see revised text). This is clearly a important difference, considering that the problem of representing three-dimensional materials science data for machine learning is indeed challenging, and the quality of the representation greatly influences model performance.
For example, it is well-known that small changes in the lattice parameters can cause large changes in the scattering angle and therefore in the powder XRD pattern, as also pointed out by the authors of Ref. 17. This raises serious doubts regarding the suitability and robustness of the powder XRD of Ref. 17 as descriptor for representing crystal structures; it also might explain the relatively poor performance of their model and why their approach fails when tested on novel structures.
A significant contribution of our work is to propose a representation that, starting from complicated and noisy three-dimensional structural data, compactly encodes detailed structural informations, while at

the same time be very robust to defect. The robustness to defects is indeed one of the main strengths of our work, as also recognized by the referee.

3.2) We disagree with the referee on the fact that crystal cubic structures are uncomplicated. As already clearly stated above (point 1.5), the starting point of our procedure are three-dimensional real-space crystal structures from which it is highly not trivial to automatically (and without having any experimental diffraction data) obtain the correct class, especially in presence of defects. This is clear from the comparison provided with a state-of-the-art method (Spglib) in Table 1, S1 and S2.

The fact that we are able to correctly classify heavily defective structures just from a single (RGB) image is actually not a drawback of our approach, but a strength: a compact, physically motivated, and easily interpretable descriptor to represent "average" long-range order in crystal structures, which allows to obtain perfect classification even in the presence of highly defective structures. This is possible because we combine diffraction patterns from different directions and use a short wavelength (~$5.0 \text{x} 10^{12}$ m) to fully exploit the systematic absences and have a large Ewald sphere, so that we can classify structure only with a single RGB image for each system.

A detailed accuracy comparison with one of the most commonly used packages for the symmetry determination (Spglib) is shown in Table 1, S1 and S2 for a total of more than 100,000 crystal structures. Our approach has an accuracy of 100% even with 25% vacancies, while Spglib has an accuracy of ~0% already at 1% vacancy concentration. The situation is similar also for random displacements. Such a large improvement is what could be defined in our opinion as a real material-science breakthrough.

We would also like to point out that our work is not only "materials-simulation relevant", but it is also relevant for all experimental techniques which produce real-space three-dimensional structural data, the most notable example being atom-probe tomography.

3.3) We have generalized our procedure to systems beyond the cubic symmetry, which now include the following space groups: 139 (body-centered-tetragonal), 141 (body-centered-tetragonal), 166 (rhombohedral), 194 (hexagonal), 221 (simple cubic), 225 (face-centered-cubic), 227 (diamond), and 229 (body-centered-cubic). These space groups cover more than 80% of the crystal structures formed in nature by elemental solids under standard conditions.

3.4) Following the referee's suggestions, we have clarified in the abstract that our goal is to classify three-dimensional structural data (and not x-ray images):

"Our study paves the way for crystal-structure recognition of – possibly noisy and incomplete - three-dimensional structural data in computational and experimental big-data materials science."

We have also explicitly stated this in "Introduction":

"Here, we propose a novel procedure to efficiently represent and classify potentially noisy and incomplete three-dimensional materials science structural data according to their crystal symmetry (and not to classify x-ray diffraction images, or powder x-ray diffraction data[17]). These three-dimensional structural data could be for example atomic structures from computational materials science databases, or elemental mappings from atom-probe tomography experiments."

Reviewers' comments:

Reviewer #1 (Remarks to the Author):

I don’t see any novel interesting point even in the revised manuscript, except for the use of CNN in the structure classification for the defect structures obtained from the database of computational materials science. I would really appreciate if the CNN was used for practical data analyses such as selected area electron diffraction (SAED) pattern, single crystal diffraction patterns, powder patterns, and so forth. The two-dimensional diffraction fingerprint obtained in this work is nothing but an arbitrary-defined feature that has no general applicability in the field. In addition, only a limited clock wise and counterclockwise rotation of 45o about the x-, y-, and z- axis would not make a representative finger print for a certain structures. What if the rotation about the [111] direction was is included as training data set ? The authors should provide readers with a rationale elucidating how the two-dimensional diffraction fingerprint could be related with some actual experimental data that is easily available in the field of materials science. My personal opinion is that the two-dimensional diffraction fingerprint may be well linked to the actual single crystal diffraction pattern or SAED pattern. I recommend the authors to reinforce the paper by including a discussion part about a real practical application for their approach.

The CNN classification achieved by the authors for simple elemental solids belonging to specific space groups exhibiting relatively high symmetries can't be treated as robust as claimed. I recommend the authors to change the tile, since it awkwardly exaggerate real contents of the paper. The present work is limitedly concerned with simple, virtual crystals that are not frequently faced with in the real functional materials world. This is in a sharp contrast to the conventional CNN-involved deep learning field, wherein a lot of CNN’s applications are emulating with existing rule-based tasks that has been known to be pretty tricky. Although the authors argued that their approach is robust since some defected structures were well identified as their parental class, it sounds irrational in terms of the general trend of deep learning. The authors’ strategy to sort out the defected structure treatment seems to me like just a well-known data augmentation. It is well known that the data augmentation could be adopted inevitably only when the size of training data set was far below than actually required.

The authors approach for defected structures appears to be irrational also in terms of practical application. For example, in case of a simple perovskite structure, a small displacement (defect) of cations at the center of cubic perovskite structure deformed to a non-centrosymmetric tetragonal structure belonging to a different class, that is in contrast to the authors claim that " it is not possible to obtain any long-range re-organization of the crystal, necessary to change the materials’ class;" This non-centrosymmetric perovskite, however, is a highly useful as a ferroelectric (piezoelectric) material, while cubic perovskite never gives any functionality but useless for the industrial application. Would it be a benefit to recognize the piezoelectric material as a cubic? Also, I’ve never seen any practical material having 20% vacancy and maintaining its parental structure.

The rebuttals are full of just a repetition of their own crooked arguments by disregarding the reviewer’s comments. This paper could be acceptable if the authors include some discussions regarding the limitation of their work by referring to the reviewers' comments. There is a small typo in the method section "we are free choose" should be "we are free to choose"

Reviewer #2 (Remarks to the Author):

The principle criticism of the original version of this work was that the data set investigated was restricted to only cubic structures. The authors have responded to this criticism by adding several additional crystal types, including tetragonal, hexagonal, and rhombohedral, which along with the

cubic systems from the original version cover the most important crystal lattice types. The model is still able to classify these structures with 100% accuracy for a hold-out set comprising 10% of the original data set, indicating that the approach is robust and generalizable to different lattice types. However, the authors should probably note that the spglib package used to create this training and test set gives different results for the space group numbers from those listed in the AFLOWLIB database where these structures are retrieved from: in fact, a quick search on aflowlib.org reveals that there are no elemental solids (i.e. materials composed of just one elemental species) that are listed as having the space group 142, for example. This is important to understand for readers who might want to try to reproduce this work by retrieving the same data set from the AFLOWLIB database.

This discrepancy in the space groups given by conventional symmetry analysis methods also raises an additional concern: how important is the accuracy of the classification of the initial training data, particularly in terms of which diffraction pattern corresponds to which space group, to the development of the model?

In addition to expanding the training and test sets, the authors have used the beta-Po prototype structure to demonstrate that the model is capable of identifying the transition between different types of lattices. This structure changes from rhombohedral to BCC, FCC and simple cubic for different lattice parameter ratios, and the model is able to correctly identify these transitions. The only question that remains is how sensitive are the classifications of model to these parameters: e.g. if the angle between the lattice vectors is very close to, but not exactly, 90 degrees, would it identify the structure as simple cubic or rhombohedral? I.e., is there an intermediate region similar to what was observed in the tests shown in Fig. 4a?

One remaining issue raised in the original report that the authors should still address is the computational demands comparison. They mention that their model takes 70ms on a specific machine to classify a structure, but they should also mention the time taken by, for example, spglib, for the same task. This would allow readers to directly compare the relative computational cost for the different methods.

Finally, there remain some typos that should be fixed, e.g. “thermodinamically” (at the start of page 5) should be “thermodynamically”.

Reviewer #3 (Remarks to the Author):

I am still favorable towards the publication of this work in Nature Communications. The recent revisions are a significant improvement. But I firmly insist that the abstract make it clear that the trial data consists of “calculated diffraction images from simulated crystal structures” rather than experimental diffraction images. They are welcome to speculate about the broader potential implications of their present work, but MUST be clear about what they have actually done to date. These details can be found in the body of the manuscript, but should be crystal clear in the abstract.

Manuscript: "The face of crystals: insightful classification using deep learning"
Reply to the Reviewers' comments

Reviewers' comments:

Reviewer #1 (Remarks to the Author):

I don't see any novel interesting point even in the revised manuscript, except for the use of CNN in the structure classification for the defect structures obtained from the database of computational materials science. I would really appreciate if the CNN was used for practical data analyses such as selected area electron diffraction (SAED) pattern, single crystal diffraction patterns, powder patterns, and so forth. The two-dimensional diffraction fingerprint obtained in this work is nothing but an arbitrary-defined feature that has no general applicability in the field. In addition, only a limited clock wise and counterclockwise rotation of 45o about the x-, y-, and z- axis would not make a representative finger print for a certain structures. What if the rotation about the [111] direction was is included as training data set ? The authors should provide readers with a rationale elucidating how the two-dimensional diffraction fingerprint could be related with some actual experimental data that is easily available in the field of materials science. My personal opinion is that the two-dimensional diffraction fingerprint may be well linked to the actual single crystal diffraction pattern or SAED pattern. I recommend the authors to reinforce the paper by including a discussion part about a real practical application for their approach.

We thank the referee for the careful reading of the revised version of the manuscript. Below we address the remaining criticisms.
The connection between two-dimensional diffraction fingerprint and experimental diffraction techniques was already mentioned in the manuscript. For example, in the section Results:
"Following the successful application of scattering concepts in determining atomic structures (using for example x-rays[30], electrons[31] or neutrons[32], we propose the diffraction pattern intensity as central quantity to describe crystal structures:"

However, to stress this link between the two-dimensional diffraction fingerprint and experimentally available techniques, we have added the following sentence in the section Methods (in blue the sentence added):

" […] Therefore, we use a wavelength of $\lambda = 5.0 \times 10^{-12}$m for the incident plane wave , a wavelength typically used in electron diffraction experiments. *Indeed, the two-dimensional diffraction fingerprint bears resemblance to experimental scattering techniques such as single-crystal or zone axis selected-area electron diffraction; from this perspective, the angle of rotation could be chosen based on specific crystal orientations[61,62].*"
[61] H. J. Bunge, Texture analysis in materials science: mathematical methods. Butterworths, 1982.
[62] T. Britton *et al.*, Materials Characterization, **117**, 113 (2016)

Regarding including different axis of rotations, one simply needs to generate a consistent set of training images, which will be then passed to the neural network. The important point here is that diffraction patterns corresponding to different classes must not accidentally became degenerate, as already stated in the section Results of the manuscript:

“Any other choice of rotation angle is in principle valid, provided that the diffraction patterns corresponding to different crystal classes do not accidentally become degenerate”

Since the neural network filters are learned directly from the data, and do not assume any prior knowledge on the type of images, the classification procedure would be exactly the same: one would just need to retrain the network with the new images obtained from the chosen rotation.

We most certainly agree that the two-dimensional diffraction fingerprint is not able to discriminate between certain structures, as already stated at point 7 in Results section. This is due to Friedel’s law and that the diffraction fingerprint is a 2D projection of a 3D diffraction pattern. This fact is highlighted even further in the new paragraph regarding the limitation of the proposed method (see below, at the end of the reply to Reviewer #1, highlighted in blue). Nevertheless, in our work we showed that we can correctly identify space groups which cover more than 80% of the crystal structures formed in nature by elemental solids under standard conditions.

Regarding the application to experimental data, we stress that our goal is to automatically classify - possible noisy and incomplete - three-dimensional structural data, for which no experimental x-ray or electron diffraction measurements are present nor possible, and not to classify x-ray diffraction images, or powder x-ray diffraction data, or electron diffraction patterns.
Experimental data that could be classified by our procedure are elemental mappings from atom-probe tomography experiment, as stated in the Introduction:
“These three-dimensional structural data could be for example atomic structures from computational materials science databases, or elemental mappings from atom-probe tomography experiments.”

The CNN classification achieved by the authors for simple elemental solids belonging to specific space groups exhibiting relatively high symmetries can't be treated as robust as claimed. I recommend the authors to change the tile, since it awkwardly exaggerate real contents of the paper. The present work is limitedly concerned with simple, virtual crystals that are not frequently faced with in the real functional materials world. This is in a sharp contrast to the conventional CNN-involved deep learning field, wherein a lot of CNN’s applications are emulating with existing rule-based tasks that has been known to be pretty tricky. Although the authors argued that their approach is robust since some defected structures were well identified as their parental class, it sounds irrational in terms of the general trend of deep learning. The authors’ strategy to sort out the defected structure treatment seems to me like just a well-known data augmentation. It is well known that the data augmentation could be adopted inevitably only when the size of training data set was far below than actually required.

While the referee labels the structures considered as “simple”, the comparison provided with a state-of-the-art method (Spglib) in Table 1, S1 and S2 shows otherwise: our approach has an accuracy of 100% even with 25% vacancies, while Spglib has an accuracy of ~0% already at 1% vacancy concentration.

The other referee’s claim
“The authors’ strategy to sort out the defected structure treatment seems to me like just a well-known data augmentation.”
is clearly wrong.
In fact, our training set consists only of pristine structures, and thus no defective structure was included in the training set. This is the exactly the opposite as data augmentation.
This fact was repeatedly stated in the manuscript, *e.g.* in the “The model performance” section:
“Since no defective structure was included at training”.

The authors approach for defected structures appears to be irrational also in terms of practical application. For example, in case of a simple perovskite structure, a small displacement (defect) of cations at the center of cubic perovskite structure deformed to a non-centrosymmetric tetragonal structure belonging to a different class, that is in contrast to the authors claim that " it is not possible to obtain any long-range re-organization of the crystal, necessary to change the materials' class;" This non-centrosymmetric perovskite, however, is a highly useful as a ferroelectric (piezoelectric) material, while cubic perovskite never gives any functionality but useless for the industrial application. Would it be a benefit to recognize the piezoelectric material as a cubic?

We have to note here that Reviewer #1 quotes only a portion of our statement regarding the long-range reorganization of the crystal, unfortunately altering in this way its meaning. The complete quote is (from Dataset subsection in section Methods):
"Finally, in the case of displacements, atoms are randomly moved about their original positions, and - due to this randomness - it is not possible to obtain any long-range re-organization of the crystal."
Thus, all atoms are randomly displaced, hence the impossibility to obtain a long-range re-organization of the crystal.

The transformation described by the referee (displacing only the cation at the center of a cubic perovskite structure) is most certainly different than displacing all atoms at random.
An example of transformation that change the crystal class is shown in Fig. 4A: there, we demonstrate that removing all central atoms indeed leads to a different crystal class, and that this transition is correctly predicted by the model.
In the model performance (from the Dataset subsection in section Methods):
"If random changes will unlikely modify a crystal class, it is however possible to apply targeted transformations in order to change a given crystal from one class to another."

We also already clearly stated (point 7 in Results) that the diffraction fingerprint is not unique across space groups; we also mentioned that *"if anomalous dispersion is neglected - a diffraction pattern is centrosymmetric, irrespective of whether or not the crystal itself has a centre of symmetry."*
Thus, by construction the diffraction fingerprint cannot distinguish between centrosymmetric and not centrosymmetric structures. This is also explicitly stated in the new paragraph regarding the limitation of the proposed method (see below, highlighted in blue).

Also, I've never seen any practical material having 20% vacancy and maintaining its parental structure. The rebuttals are full of just a repetition of their own crooked arguments by disregarding the reviewer's comments. This paper could be acceptable if the authors include some discussions regarding the limitation of their work by referring to the reviewers' comments. There is a small typo in the method section "we are free choose" should be "we are free to choose"

From Table 1, S1 and S2, one can see that the Spglib accuracy is ~0.0%, already at 1% of vacancies, well before the 20% concentration quoted by the referee.

We agree with the referee that arguably no material with 20% of vacancies will maintain its parental structure. However, we would like to point out that "vacancies" in a given atomic structure could also just be missing data, due for example to technical constraints in experimental measurements. In this sense, 20% of missing atoms is indeed realistic. For example, in atom-probe tomography (arguably the most important source of local atomic structure information in bulk systems) the maximum detector

efficiency is 80%, which means that at least 20% of the atoms – even though present in the actual experimental sample - are not experimentally detected, and therefore they are missing data.

As suggested by the referee, a paragraph discussing the limitation of our work has been added in the Discussion section:
"Further work is needed to make the approach proposed here unique across space groups and to widen its domain of applicability to non-centrosymmetric crystals, which can exhibit technologically relevant ferroelectric, piezoelectric or nonlinear optical effects."

The typo regarding "we are free to choose" is now corrected.

Reviewer #2 (Remarks to the Author):

The principle criticism of the original version of this work was that the data set investigated was restricted to only cubic structures. The authors have responded to this criticism by adding several additional crystal types, including tetragonal, hexagonal, and rhombohedral, which along with the cubic systems from the original version cover the most important crystal lattice types. The model is still able to classify these structures with 100% accuracy for a hold-out set comprising 10% of the original data set, indicating that the approach is robust and generalizable to different lattice types. However, the authors should probably note that the spglib package used to create this training and test set gives different results for the space group numbers from those listed in the AFLOWLIB database where these structures are retrieved from: in fact, a quick search on aflowlib.org reveals that there are no elemental solids (i.e. materials composed of just one elemental species) that are listed as having the space group 142, for example. This is important to understand for readers who might want to try to reproduce this work by retrieving the same data set from the AFLOWLIB database.

This discrepancy in the space groups given by conventional symmetry analysis methods also raises an additional concern: how important is the accuracy of the classification of the initial training data, particularly in terms of which diffraction pattern corresponds to which space group, to the development of the model?

We thank the referee for its in-depth reading of the revised version of the manuscript, and for providing constructive criticisms. Below we address the remaining points that were raised.

Calculations with space group 141 (and not 142) were considered in our work.
Unfortunately, there was a typo in the manuscript, and 142 was reported instead of 141 (Fig. 2 and the previous reply to the referees' correctly quoted 141). This is the reason for the mismatch found by the referee, which we thank for spotting this. A search on the AFLOWLIB database for structures having space group 141 (the correct one) will indeed return results, as expected.

Having a correct training set is of paramount importance for model prediction reliability. To this end, we checked that the training (pristine) dataset classification provided by Spglib is stable with respect to changes in threshold parameters. In particular, the following combination of Spglib parameters:
- `symprec`=1×10^{-3}Å, `angle_tolerance`=1°
- `symprec`=1×10^{-6}Å, `angle_tolerance`=1°
- `symprec`=1×10^{-3}Å, `angle_tolerance`=0.1°
- `symprec`=1×10^{-6}Å, `angle_tolerance`=0.1°

always gave the same classification and therefore we are confident that – for pristine structures – Spglib provides the correct "true labels" as training set for the machine learning model.

Regarding the reproducibility of our results, we provide both the raw data and the processed data.

The raw data, used as starting point for our analysis, can be freely downloaded from the NOMAD Archive; we have added a section "Links to the pristine dataset" to the Supplemental materials with the corresponding uniform resource locators (URLs).

Moreover, we have uploaded a dataset to the Harvard Dataverse (https://dataverse.harvard.edu/privateurl.xhtml?token=a3d3282f-3541-4787-b506-9bc231dded5b) which contains, for each structure considered:

1) a file with the xyz coordinates
2) a file with additional details such as NOMAD unique identifier, space group classification according to Spglib and simulation cell
3) the corresponding two-dimensional diffraction fingerprint as png image

In addition to expanding the training and test sets, the authors have used the beta-Po prototype structure to demonstrate that the model is capable of identifying the transition between different types of lattices. This structure changes from rhombohedral to BCC, FCC and simple cubic for different lattice parameter ratios, and the model is able to correctly identify these transitions. The only question that remains is how sensitive are the classifications of model to these parameters: e.g. if the angle between the lattice vectors is very close to, but not exactly, 90 degrees, would it identify the structure as simple cubic or rhombohedral? I.e., is there an intermediate region similar to what was observed in the tests shown in Fig. 4a?

To answer the referee's question, we have generated a larger number of prototypes (160) with order parameter $\mu \in [\sqrt{(3/2)}, 5\sqrt{(3/2)}]$; this results in a resolution of $\Delta\mu \approx 0.015$. The updated result is shown in Fig4b.

In particular, regarding the transition from simple cubic to rhombohedral, from Fig.4b we can see that structures having the following values for the order parameters:

μ = sqrt(3/2) ± 0.075 or $\alpha = (90 \pm 2.4)°$

are identified as simple cubic.

For example, β-Po has parameters: [R1, R2]

$\mu = 0.96814$ or $\alpha \approx 98.2°$

and thus it is identified as belonging to the hex/rh class. We do not observe any intermediate regions in this case.

[R1] Aflowlib.org: http://www.aflowlib.org/CrystalDatabase/A_hR1_166_a.beta-Po.html,
[R2] W. H. Beamer and C. R. Maxwell, J. Chem. Phys. **17**, 1293 [2] 1298 (1949)

One remaining issue raised in the original report that the authors should still address is the computational demands comparison. They mention that their model takes 70ms on a specific machine to classify a structure, but they should also mention the time taken by, for example, spglib, for the same task. This would allow readers to directly compare the relative computational cost for the different methods.

According to our computational experiments, Spglib computational cost greatly varies with the number of atoms of the simulation cell and, to a lesser extend, with the type of crystal structure. We found that prediction takes between 1ms and 10ms for simulation cells containing less than 20 atoms. For simulation cells containing ~500 atoms, prediction takes between 500ms and 700ms, depending on the crystal structure type.

A detailed investigation of the Spglib computational cost is beyond the scope of the present work, and, given the variability with respect to simulation cell size, we would rather avoid to mention any speed comparison in the manuscript. The purpose of our work in fact is not to be faster than Spglib, but to be predictive on defective structures without having to guess (or find with trial and error thus effectively increasing the computational cost) a threshold.

Finally, there remain some typos that should be fixed, e.g. “thermodinamically” (at the start of page 5) should be “thermodynamically”.

The typo was corrected.

Reviewer #3 (Remarks to the Author):

I am still favorable towards the publication of this work in Nature Communications. The recent revisions are a significant improvement. But I firmly insist that the abstract make it clear that the trial data consists of “calculated diffraction images from simulated crystal structures” rather than experimental diffraction images. They are welcome to speculate about the broader potential implications of their present work, but MUST be clear about what they have actually done to date. These details can be found in the body of the manuscript, but should be crystal clear in the abstract.

We thank the referee for his/her comments. As requested, we have modified the abstract to make absolutely clear that “calculated diffraction images from simulated crystal structures” were used in our work.
Here we report the section of the abstract that was modified (in blue the modifications):
“First, we represent crystals by ***calculating a*** *diffraction image, then construct a deep-learning neural-network model for classification. Our approach is able to correctly classify a dataset comprising more than 100,000* ***simulated*** *crystal structures, including heavily defective ones.”*

Reviewers' comments:

Reviewer #2 (Remarks to the Author):

Since Reviewer #1 was unable to provide a report on the revised version, the editors requested that I (Reviewer #2) evaluate the response to the technical concerns raised by this reviewer, as well as to my own comments.

Evaluation of reply to Reviewer #1:

Reviewer #1 had several criticisms of the work. The first criticism is the reviewer’s claim that there do not appear to be any major applications of this method. This may stem from the Reviewer’s own unfamiliarity with computational methodology, where automatically identifying the correct symmetry properties of a set of atomic coordinates and lattice vectors is a major concern. Important computational applications of symmetry include constructing appropriate k-point grids and generating the correct path between the high-symmetry points for band structure calculations; and for identifying the appropriate distortions for finite-displacement phonon calculations. Due to tolerance issues with noisy experimental or computational data used as the starting point for first-principles calculations, the standard approach of identifying which symmetry operations are satisfied is non-trivial, making this machine-learning approach interesting. However, the authors should probably explicitly mention some of these specific applications in the manuscript, which will increase its accessibility and put the work into context for a more general audience.

The second issue raised by Reviewer #1 regards the rotation angles used to produce and analyze the different diffraction patterns. Since the method is intended to be applied to crystal structures in the form of sets of atomic coordinates and lattice vectors, the users of the code can control rotation angles and crystal orientations, allowing them to choose these angles. Therefore, the fact that the method requires certain rotations should not be an issue. However, this does raise the question of how important the choice of a standard initial crystal orientation is for this method. The authors should clarify if they always pre-align the crystal in a certain orientation prior to generating the diffraction image, and confirm that the method is robust against randomization of the initial crystal orientations. In particular, the authors should provide some more detail on how they generate and orientate the conventional cell, since they claim to follow the approach of Setyawan and Curtarolo (Ref. 33 in their manuscript) to generate this cell. However, the version of this approach implemented in the AFLOW code depends on the AFLOW symmetry routines (see arXiv:1802.07977). If traditional symmetry analysis routines are required to prepare the input files for the neural network, then this would seem to defeat the purpose of the new machine-learning method, since its results would still be dependent on the reliability of the traditional symmetry approach. The authors need to address this point clearly, to clarify exactly what methods are used to generate the standard conventional cells, and demonstrate that their method is not dependent on, or is not affected by, the use of standard symmetry routines to prepare the input data.

The third point raised by the Reviewer involves the fact that the method classifies structures with (small) random displacements as being of the “parent” crystal class, claiming that this would remove the distinction between, for example, the centrosymmetric cubic perovskite and the non-centrosymmetric tetragonal perovskite. However, this cubic to tetragonal shift is not random; instead, the displacements of all of the atoms in the crystal sample would be highly correlated. The authors do acknowledge that there is a limitation of their model when it comes to distinguishing between centrosymmetric and non-centrosymmetric structures, and have added a comment to the manuscript to clarify this. However, it is strange that they seem to regard this as an advantage of the approach, since in item 7 on page 4 they list “not unique across space groups” as one of the “desirable properties” of the method; this would seem to be more of a limitation than an advantage.

The fourth point raised by the Reviewer concerns the example where up to 20% of atoms are removed from the crystal structure, where the method is still able to identify the parent crystal class. The Reviewer is correct in that in reality, a structure missing 20% of its atoms would no longer be stable. However, the authors correctly respond that in practice, experimental structure characterization techniques regularly fail to detect a large proportion of atoms, thus rendering this feature useful.

Another point raised by the Reviewer is that the generation of sample structures by applying various distortions or removing atoms could be considered as “data augmentation”. However, the authors correctly respond that since the training set only contains pristine structures, and that the defected structures are used purely for testing, then this should not be an issue.

Finally, Reviewer #1 raises a concern about the title of this work, in that it could mislead readers into believing that the developed model is more general and powerful than it actually is. This concern appears to be also shared somewhat by Reviewer #3, who correctly insists on a rewording of the abstract. The authors should consider a more specific title for this work, which more accurately reflects its contents and the limitations of the model. In particular, they need to make clear they start from a crystal structure (i.e. lattice vectors and atomic coordinates), which they then used to generate a diffraction image that is analyzed using the neural network.

Additional comments from Reviewer #2:

The authors have corrected the issues with the space group mismatch with the AFLOWLIB database, and have verified the spglib characterization of the training set by using different tolerance parameters, which is important since the default spglib tolerances have been shown to be problematic (see, e.g., arXiv:1802.07977). They have also refined the investigation of the model’s performance at identifying the structure class of a material as it is continuously distorted, providing additional detail as to the effective tolerances and sensitivity of the model.

For the computational cost issue, they explain that the time can vary for the traditional methods depending in the structure being investigated. I recommend that they add this information to the manuscript, as it will help readers to assess the relative strengths and weaknesses of the different approaches.

Finally, as noted above, Reviewer #1’s questions about the rotation angles/axes used to generate the diffraction images highlighted an issue regarding the generation of the standard conventional cell used to create the diffraction patterns. The authors state that they are following the approach of Setyawan and Curtarolo (Ref. 33 in their paper) to find the correct conventional cell. However, this approach, as implemented in the AFLOW code, depends on standard symmetry routines based around applying rotation and translation operations to coordinates in order to find the correct transformations required to build the standard conventional cell and the supercells (see arXiv:1802.07977). Depending on exactly how they implement this approach, this could mean that the robustness or accuracy of any analysis they perform would still be dependent on the traditional symmetry routines. The authors need to provide more detail about the exact approach used to generate the standard conventional cells and supercells, such as whether these cells are generated prior to the introduction of the defects (i.e. removal or random displacement of atoms) so that the modifications are applied to an already generated supercell, or whether supercells are being generated for the defect cells. The authors need to demonstrate that the reliability of their method is independent of the use of standard symmetry approaches in formatting the input data, since this could be a major limiting factor for this approach.

Manuscript: "Insightful classification of crystal structures using deep learning"
Reply to the Reviewers' comments

We thank Referee #2 for taking the time to carefully read our revised manuscript, for his/her detailed and constructive criticisms which improved our work, and for evaluating also our reply to Referee #1. Below we address his/her last remaining points.

Reviewers' comments:

Evaluation of reply to Reviewer #1:

Reviewer #1 had several criticisms of the work. The first criticism is the reviewer's claim that there do not appear to be any major applications of this method. This may stem from the Reviewer's own unfamiliarity with computational methodology, where automatically identifying the correct symmetry properties of a set of atomic coordinates and lattice vectors is a major concern. Important computational applications of symmetry include constructing appropriate k-point grids and generating the correct path between the high-symmetry points for band structure calculations; and for identifying the appropriate distortions for finite-displacement phonon calculations. Due to tolerance issues with noisy experimental or computational data used as the starting point for first-principles calculations, the standard approach of identifying which symmetry operations are satisfied is non-trivial, making this machine-learning approach interesting. However, the authors should probably explicitly mention some of these specific applications in the manuscript, which will increase its accessibility and put the work into context for a more general audience.

1) We thank Ref #2 for his/her pertinent suggestion; thus, we have added the following paragraph to the introduction:
"From the computational point of view, automatic identification of crystal symmetries allows for example to construct appropriate k-point grids for Brillouin zone sampling, to generate path between high-symmetry points in band structure calculations, or to identify distortions for finite-displacement phonon calculations."
to highlight additional applications of our approach in computational methodologies.

The second issue raised by Reviewer #1 regards the rotation angles used to produce and analyze the different diffraction patterns. Since the method is intended to be applied to crystal structures in the form of sets of atomic coordinates and lattice vectors, the users of the code can control rotation angles and crystal orientations, allowing them to choose these angles. Therefore, the fact that the method requires certain rotations should not be an issue. However, this does raise the question of how important the choice of a standard initial crystal orientation is for this method. The authors should clarify if they always pre-align the crystal in a certain orientation prior to generating the diffraction image, and confirm that the method is robust against randomization of the initial crystal orientations. In particular, the authors should provide some more detail on how they generate and orientate the conventional cell, since they claim to follow the approach of Setyawan and Curtarolo (Ref. 33 in their manuscript) to

generate this cell. However, the version of this approach implemented in the AFLOW code depends on the AFLOW symmetry routines (see arXiv:1802.07977). If traditional symmetry analysis routines are required to prepare the input files for the neural network, then this would seem to defeat the purpose of the new machine-learning method, since its results would still be dependent on the reliability of the traditional symmetry approach. The authors need to address this point clearly, to clarify exactly what methods are used to generate the standard conventional cells, and demonstrate that their method is not dependent on, or is not affected by, the use of standard symmetry routines to prepare the input data.

2) We thank the referee for pointing this out. In the previous version of the manuscript, we generated the conventional cell using pymatgen (http://pymatgen.org/pymatgen.symmetry.analyzer.html#pymatgen.symmetry.analyzer.SpacegroupAnalyzer.get_conventional_standard_structure)
The referee is indeed correct that symmetries are used in the pymatgen implementation of the conventional cell construction; we also agree that if this influences the results, it would definitely constitute a drawback of the proposed method.

To tackle this, we slightly modify our procedure in order to make it completely independent from traditional symmetry analysis routines. In particular, we use the convention for triclinic cells (the most general lattice) irrespectively of the actual lattice type of the crystal structure, and we do not apply any symmetry refinement of the atomic position.
The actual snippet of Python code used to generate the standard conventional cell in our work is presented in the "Appendix: Conventional Standard Cell Python Implementation" at the end of this document.
This code is essentially the pymatgen implementation of the conventional cell for triclinic lattices (http://pymatgen.org/_modules/pymatgen/symmetry/analyzer.html#SpacegroupAnalyzer.get_conventional_standard_structure), as specified in Setyawan and Curtarolo [Ref. 33 in the manuscript], but without using refinement of atomic positions.

To prove the robustness with respect to initial crystal orientation, we concatenate three random rotations around the three crystal axes prior to the calculation of the conventional cell for each structure in the dataset. Furthermore, we have also tested that application of these random rotations followed by the conventional cell determination on already generated (defective) supercells leads to the same result.

This (updated) procedure is now completely independent from traditional symmetry analysis routines and proven to be robust with respect to randomization of the initial crystal orientation. This is explained in detail in the Methods/Two-dimensional diffraction fingerprint section of the revised manuscript:

"*First, for each structure in the dataset (specified by a set of atomic coordinates and lattice vectors), we concatenate three random rotations around the three crystal axes to randomize the initial crystal orientation. Then, we construct the standard conventional cell according to Ref.33 using a customized implementation based on the Python Materials Genomics (pymatgen) package[60]; in particular, we use the convention for triclinic cells - irrespective of the actual lattice type - and no symmetry refinement of the atomic position. This procedure is therefore completely independent from traditional symmetry approaches and robust against randomization of the initial crystal orientation. Finally, we replicate*

this standard cell in all three directions such that the resulting cluster contains a number of atoms which is as close as possible to a given target number (namely, 250). The size-invariance of the diffraction peak locations guarantees that the results are independent from this choice, only the peak widths will slightly change, in accordance with the indetermination principle (this was expressly checked for systems ranging from 32 to 1024 atoms). Defective structures are then generated from these supercells removing or randomly displacing atoms. We have also tested that a random rotation followed by the conventional cell determination applied to already generated defective structures leads to the same result, since this depends on the lattice vectors only."

From the crystal structures generated from this (updated) procedure, we calculate diffraction images, and train a neural network with these new images using the same architecture as before (see Section: Methods/Neural network architecture and training procedure). We then use this neural network to predict the quantities reported in the manuscript. The main results presented in the manuscript are not altered in any way by these modifications; in particular the only changes are:

2a) Fig. 2c and Fig. 5b: The diffraction pattern for the rh/hex class is modified; we have updated accordingly the images for the hex/rh class in Fig. 2c (two-dimensional diffraction fingerprint) and Fig. 5b (attentive response map).

2b) Fig.4a: The body-centered-cubic (bcc) to simple-cubic structural transition plot was recalculated with the neural network trained on the new images. The only difference is that the bcc classification probability (red line in Fig. 4a) starts decreasing at 75% instead of 70% obtained with the previous neural network; the number in the text was updated accordingly (in blue):
"*However, at* ***75****% of central atoms removed*"

2c) Fig. 4b: We show that our approach is again able to identify when the prototype reduces to the high-symmetry structures (bcc α=109.47˚, sc α=90˚, and fcc α=60˚) and also correctly classify the structure as belonging to the hex/rh class for all other values of α.
The only difference is that now the rhombohedral structure is recognized as simple cubic exactly at α=90˚, while before structures within a small interval around α=90˚ ($\alpha \in$ (87.6°, 92.4°)) were also classified as simple cubic. The previous recognition of rhombohedral structure as simple cubic for $\alpha \in$ (87.6°, 92.4°) was due to the refinement of the atomic positions which is now removed.

2d)
The accuracy in identifying the correct crystal class in the presence of defects were recalculated with the neural network trained on the new images. We observe only very small changes (highlighted in blue) in the accuracy. We copy below the current Table S1 and S2:

	Random Displacements (σ)										
	0.001Å	0.002Å	0.003Å	0.004Å	0.005Å	0.01Å	0.02Å	0.04Å	0.06Å	0.08Å	0.10Å
Spglib (tight)	0.00	0.00	0.00	0.00	0.00	0.00	0.00	0.00	0.00	0.00	0.00
Spglib (medium)	73.70	0.00	0.00	0.00	0.00	0.00	0.00	0.00	0.00	0.00	0.00
Spglib (loose)	99.99	99.99	99.99	99.99	99.99	75.22	0.00	0.00	0.00	0.00	0.00
**This work**	100.00	100.0	100.00	100.00	100.00	100.00	100.00	100.00	100.00	99.97	99.91

Table S1. Accuracy in identifying the correct ("most similar") crystal class in the presence of defects. The defective structures are calculated randomly displacing atoms according to Gaussian distribution with standard deviation σ. The accuracy values are in percentage.

	Vacancies (η)											
	1 %	2 %	5 %	10 %	15 %	20 %	25 %	30 %	40 %	50 %	60 %	70 %
Spglib (tight)	0.02	0.00	0.00	0.00	0.00	0.00	0.00	0.00	0.00	0.00	0.00	0.00
Spglib (medium)	0.02	0.00	0.00	0.00	0.00	0.00	0.00	0.00	0.00	0.00	0.00	0.00
Spglib (loose)	0.01	0.00	0.00	0.00	0.00	0.00	0.00	0.00	0.00	0.00	0.00	0.00
**This work**	100.00	100.00	100.00	100.00	100.00	100.00	100.00	100.00	100.00	99.56	97.18	89.05

Table S2. Accuracy in identifying the correct ("most similar") crystal class in the presence of defects. The defective structures are calculated randomly removing η% of the atoms, thus creating vacancies. The accuracy values are in percentage.

For convenience, we copy here the previous version of the same tables:

	Random Displacements (σ)										
	0.001Å	0.002Å	0.003Å	0.004Å	0.005Å	0.01Å	0.02Å	0.04Å	0.06Å	0.08Å	0.10Å
Spglib (tight)	0.00	0.00	0.00	0.00	0.00	0.00	0.00	0.00	0.00	0.00	0.00
Spglib (medium)	73.70	0.00	0.00	0.00	0.00	0.00	0.00	0.00	0.00	0.00	0.00
Spglib (loose)	99.99	99.99	99.99	99.99	99.99	75.22	0.00	0.00	0.00	0.00	0.00
**This work**	100.00	100.0	100.00	100.00	100.00	100.00	100.00	100.00	100.00	100.00	99.99

Table S1. Accuracy in identifying the correct ("most similar") crystal class in the presence of defects. The defective structures are calculated randomly displacing atoms according to Gaussian distribution with standard deviation σ. The accuracy values are in percentage.

	Vacancies (η)											
	1 %	2 %	5 %	10 %	15 %	20 %	25 %	30 %	40 %	50 %	60 %	70 %
Spglib (tight)	0.02	0.00	0.00	0.00	0.00	0.00	0.00	0.00	0.00	0.00	0.00	0.00
Spglib (medium)	0.02	0.00	0.00	0.00	0.00	0.00	0.00	0.00	0.00	0.00	0.00	0.00
Spglib (loose)	0.01	0.00	0.00	0.00	0.00	0.00	0.00	0.00	0.00	0.00	0.00	0.00
**This work**	100.00	100.00	100.00	100.00	100.00	100.00	100.00	99.99	99.97	99.56	98.32	71.74

Table S2. Accuracy in identifying the correct ("most similar") crystal class in the presence of defects. The defective structures are calculated randomly removing η% of the atoms, thus creating vacancies. The accuracy values are in percentage.

Table 1 (which is a subset of Table S1 and S2), together with the values mentioned in the text have been updated accordingly (changes highlighted in blue in the manuscript).

We stress the fact that none of the conclusion of the manuscript have changed.

The third point raised by the Reviewer involves the fact that the method classifies structures with (small) random displacements as being of the "parent" crystal class, claiming that this would remove the distinction between, for example, the centrosymmetric cubic perovskite and the non-centrosymmetric tetragonal perovskite. However, this cubic to tetragonal shift is not random; instead, the displacements of all of the atoms in the crystal sample would be highly correlated. The authors do acknowledge that there is a limitation of their model when it comes to distinguishing between centrosymmetric and non-centrosymmetric structures, and have added a comment to the manuscript to clarify this. However, it is strange that they seem to regard this as an advantage of the approach, since in item 7 on page 4 they list "not unique across space groups" as one of the "desirable properties" of the method; this would seem to be more of a limitation than an advantage.

3) We thank the referee for correctly pointing out this typo. Indeed, the non uniqueness is a disadvantage of our representation. We removed it from the list of "desirable properties" and added it just below the list, as a disadvantage (see modified text in blue):
"*A disadvantage of the two-dimensional diffraction fingerprint is that it is not unique across space groups. [...]*"

The fourth point raised by the Reviewer concerns the example where up to 20% of atoms are removed from the crystal structure, where the method is still able to identify the parent crystal class. The Reviewer is correct in that in reality, a structure missing 20% of its atoms would no longer be stable. However, the authors correctly respond that in practice, experimental structure characterization techniques regularly fail to detect a large proportion of atoms, thus rendering this feature useful.

Another point raised by the Reviewer is that the generation of sample structures by applying various distortions or removing atoms could be considered as "data augmentation". However, the authors correctly respond that since the training set only contains pristine structures, and that the defected structures are used purely for testing, then this should not be an issue.

Finally, Reviewer #1 raises a concern about the title of this work, in that it could mislead readers into believing that the developed model is more general and powerful than it actually is. This concern appears to be also shared somewhat by Reviewer #3, who correctly insists on a rewording of the abstract. The authors should consider a more specific title for this work, which more accurately reflects its contents and the limitations of the model. In particular, they need to make clear they start from a crystal structure (i.e. lattice vectors and atomic coordinates), which they then used to generate a diffraction image that is analyzed using the neural network.

4) The choice of the title "*The face of crystals: insightful classification using deep learning*" was motivated by the fact that we represent crystal structures by images, and use the same computational

models (convolutional neural networks) used in computer vision to classify natural images (*e.g.* human faces).
Nevertheless, to meet the referee's request, we changed the title of the manuscript to:
"*Insightful classification of crystal structures using deep learning*"
Moreover, as suggested by the Referee, we specify that we start from a set of coordinate and lattice vectors (see additions in blue) both in the Introduction
"However, our goal here is to introduce an automatic procedure to classify crystal structures starting from a set of atomic coordinates and lattice vectors"
and in Section Methods/Two-dimensional diffraction fingerprint:
"First, for each structure in the dataset (specified by a set of atomic coordinates and lattice vectors) [...]"

This concern appears to be also shared somewhat by Reviewer #3, who correctly insists on a rewording of the abstract.
5) Regarding this sentence, we think that Ref #3 meant "insisted" and not "insists", since we already addressed this in the previous revised manuscript (sent Feb 22nd, 2018).
For reference we copy here the last comment from Reviewer #3 in the previous round of review (Jan 23rd, 2018), and our reply in the previous revision of the manuscript (sent Feb 22nd, 2018):

"[LAST COMMENT FOR REFEREE #3 (Jan 23rd, 2018)]
I am still favorable towards the publication of this work in Nature Communications. The recent revisions are a significant improvement. But I firmly insist that the abstract make it clear that the trial data consists of "calculated diffraction images from simulated crystal structures" rather than experimental diffraction images. They are welcome to speculate about the broader potential implications of their present work, but MUST be clear about what they have actually done to date. These details can be found in the body of the manuscript, but should be crystal clear in the abstract."
Thus, Referee #3 only asked to specify – also in the abstract – that we use calculated diffraction images, and not experimental diffraction images.

[AUTHORS' REPLY ON FEB 22, 2018]
We thank the referee for his/her comments. As requested, we have modified the abstract to make absolutely clear that "calculated diffraction images from simulated crystal structures" were used in our work.
Here we report the section of the abstract that was modified (in blue the modifications):
"First, we represent crystals by calculating a diffraction image, then construct a deep-learning neural-network model for classification. Our approach is able to correctly classify a dataset comprising more than 100,000 simulated crystal structures, including heavily defective ones."

Additional comments from Reviewer #2:

The authors have corrected the issues with the space group mismatch with the AFLOWLIB database, and have verified the spglib characterization of the training set by using different tolerance parameters, which is important since the default spglib tolerances have been shown to be problematic (see, e.g.,

arXiv:1802.07977). They have also refined the investigation of the model's performance at identifying the structure class of a material as it is continuously distorted, providing additional detail as to the effective tolerances and sensitivity of the model. For the computational cost issue, they explain that the time can vary for the traditional methods depending in the structure being investigated. I recommend that they add this information to the manuscript, as it will help readers to assess the relative strengths and weaknesses of the different approaches.

7) As stated in the previous round of review, a detailed investigation of the Spglib computational cost is beyond the scope of the present work, and we would rather avoid to mention any speed comparison in the manuscript. The purpose of our work in fact is not to be faster than Spglib, but to be predictive on defective structures without having to guess a threshold, or find it with trial and error thus effectively increasing the computational cost. A fair comparison would require to express these considerations in a somewhat quantitative manner, in addition to extensively optimize and benchmark our code and associated libraries (*e.g.* Condor [Ref. 64] for the diffraction images generation), which is beyond the scope of the present paper.

Finally, as noted above, Reviewer #1's questions about the rotation angles/axes used to generate the diffraction images highlighted an issue regarding the generation of the standard conventional cell used to create the diffraction patterns. The authors state that they are following the approach of Setyawan and Curtarolo (Ref. 33 in their paper) to find the correct conventional cell. However, this approach, as implemented in the AFLOW code, depends on standard symmetry routines based around applying rotation and translation operations to coordinates in order to find the correct transformations required to build the standard conventional cell and the supercells (see arXiv:1802.07977). Depending on exactly how they implement this approach, this could mean that the robustness or accuracy of any analysis they perform would still be dependent on the traditional symmetry routines. The authors need to provide more detail about the exact approach used to generate the standard conventional cells and supercells, such as whether these cells are generated prior to the introduction of the defects (i.e. removal or random displacement of atoms) so that the modifications are applied to an already generated supercell, or whether supercells are being generated for the defect cells. The authors need to demonstrate that the reliability of their method is independent of the use of standard symmetry approaches in formatting the input data, since this could be a major limiting factor for this approach.

8) We addressed this question at Point **2)** of this document.

Other minor changes (highlighted in blue in the manuscript):

9) To make absolutely sure that Spglib thresholds are not influencing our results, we extract structures that have a consistent space group across a large range of Spglib `symprec` thresholds; this is explained in Methods/Dataset:

"*Specifically, we extract structures that have a consistent space group classification for different symmetry tolerances, as determined by the Python Materials Genomics (pymatgen)[60] wrapper around the Spglib[11] library with* `symprec`*={10*$^{-3}$*Å, 10*$^{-6}$*Å, 10*$^{-9}$*Å} for all except rh and hex structures, for which* `symprec`*={10*$^{-3}$*Å, 10*$^{-6}$*Å} is employed since some symmetries are missed for* `symprec`*=10*$^{-9}$*Å}.*"

The pristine (defective) dataset now contains 10 517 (105 170) structures, instead of 10 765 (107 650).

10) In Results (How to represent a material: the descriptor):

"*This procedure is then repeated for all three crystal axis.*"

with

"*This procedure is then repeated for all three crystal ax**e**s.*"

11) Updated reference to AFLOW-SYM (Ref. 12) since now the work is available on the ArXiv (1802.07977).

Appendix: Conventional Standard Cell Python Implementation

```
def get_conventional_std_cell_no_sym(atoms):
    """Given an ASE atoms object, return the ASE atoms object in the conventional standard cell.

    It does NOT use symmetries to obtain the standard cell.
    Gives a structure with a conventional cell according to the standard defined for TRICLINIC cells in
    W. Setyawan, and S. Curtarolo, Comput. Mater. Sci.49(2), 299-312 (2010).
    The triclinic convention is employed to make sure that no information on the symmetry of the lattice
    is used in the generation of the standard cell.
    The code is taken from the triclinic cell case in pymatgen:
    http://pymatgen.org/_modules/pymatgen/symmetry/analyzer.html

    Parameters:
    atoms: `ase.Atoms` object
        Atomic structure.
    Returns:
    `ase.Atoms` object
        Return the structure in a conventional cell (the convention used is the one for triclinic cells)
    """
    # save atoms.info dict otherwise it gets lost in the conversion
    atoms_info = atoms.info
    mg_structure = AseAtomsAdaptor.get_structure(atoms)
    finder = SpacegroupAnalyzer(mg_structure)

    # get structure in the spglib format
    # according to the Spglib documentation
    # (https://atztogo.github.io/spglib/python-spglib.html?highlight=standardize_cell#standardize-cell)
    # ‘no_idealize=True’ disables to idealize lengths and angles of basis vectors and positions of atoms according to crystal symmetry.
    # the structure is not refined and thus symmetries are not used
    lattice, scaled_positions, numbers = spglib.standardize_cell(finder._cell, to_primitive=False, no_idealize=True)
    species = [finder._unique_species[i - 1] for i in numbers]
    struct = Structure(lattice, species, scaled_positions).get_sorted_structure()

    # this is the convention for triclinic cells in pymatgen
    # see http://pymatgen.org/_modules/pymatgen/symmetry/analyzer.html
    latt = struct.lattice
    a, b, c = latt.lengths_and_angles[0]
    alpha, beta, gamma = [math.pi * i / 180 for i in latt.lengths_and_angles[1]]
    new_matrix = None
    transf = None
    test_matrix = [[a, 0, 0], [b * cos(gamma), b * sin(gamma), 0.0],
                   [c * cos(beta), c * (cos(alpha) - cos(beta) * cos(gamma)) / sin(gamma), c * math.sqrt(
                       sin(gamma) ** 2 - cos(alpha) ** 2 - cos(beta) ** 2 + 2 * cos(alpha) * cos(beta) * cos(
                           gamma)) / sin(gamma)]]

    def is_all_acute_or_obtuse(m):
        recp_angles = np.array(Lattice(m).reciprocal_lattice.angles)
        return np.all(recp_angles <= 90) or np.all(recp_angles > 90)

    if is_all_acute_or_obtuse(test_matrix):
        transf = np.eye(3)
        new_matrix = test_matrix

    test_matrix = [[-a, 0, 0], [b * cos(gamma), b * sin(gamma), 0.0],
                   [-c * cos(beta), -c * (cos(alpha) - cos(beta) * cos(gamma)) / sin(gamma), -c * math.sqrt(
                       sin(gamma) ** 2 - cos(alpha) ** 2 - cos(beta) ** 2 + 2 * cos(alpha) * cos(beta) * cos(
                           gamma)) / sin(gamma)]]

    if is_all_acute_or_obtuse(test_matrix):
        transf = [[-1, 0, 0], [0, 1, 0], [0, 0, -1]]
        new_matrix = test_matrix

    test_matrix = [[-a, 0, 0], [-b * cos(gamma), -b * sin(gamma), 0.0],
                   [c * cos(beta), c * (cos(alpha) - cos(beta) * cos(gamma)) / sin(gamma), c * math.sqrt(
                       sin(gamma) ** 2 - cos(alpha) ** 2 - cos(beta) ** 2 + 2 * cos(alpha) * cos(beta) * cos(
                           gamma)) / sin(gamma)]]
```

```
if is_all_acute_or_obtuse(test_matrix):
    transf = [[-1, 0, 0], [0, -1, 0], [0, 0, 1]]
    new_matrix = test_matrix

test_matrix = [[a, 0, 0], [-b * cos(gamma), -b * sin(gamma), 0.0],
               [-c * cos(beta), -c * (cos(alpha) - cos(beta) * cos(gamma)) / sin(gamma), -c * math.sqrt(
                   sin(gamma) ** 2 - cos(alpha) ** 2 - cos(beta) ** 2 + 2 * cos(alpha) * cos(beta) * cos(
                       gamma)) / sin(gamma)]]

if is_all_acute_or_obtuse(test_matrix):
    transf = [[1, 0, 0], [0, -1, 0], [0, 0, -1]]
    new_matrix = test_matrix

latt = Lattice(new_matrix)
new_coords = np.dot(transf, np.transpose(struct.frac_coords)).T
new_struct = Structure(latt, struct.species_and_occu, new_coords, site_properties=struct.site_properties,
                       to_unit_cell=True).get_sorted_structure()

# put the results back in an ASE structure
conventional_standard_atoms = AseAtomsAdaptor.get_atoms(new_struct)
conventional_standard_atoms.info = atoms_info

return conventional_standard_atoms
```

REVIEWERS' COMMENTS:

Reviewer #2 (Remarks to the Author):

The main concern raised in the previous report was extent to which the machine learning method introduced in this work is dependent on the use of traditional symmetry routines to generate the conventional cell. To address this issue, the authors regenerated the cells using the convention for triclinic symmetry, meaning that the cell generation is independent of its symmetry classification, and also randomized the cell orientations. This approach made little difference to the results (only small quantitative differences in Fig. 4, and some changes to the diffraction pattern for hex and rhl systems in Figs. 2 and 5), successfully demonstrating that their approach does not depend on the routines used to generate the conventional cell.

Other minor suggestions in the previous report included modifying the title and adding some specific example applications to the introduction, both of which have been made appropriately. The authors have also fixed the list of advantages of the method to clarify that the non-uniqueness across space groups is a disadvantage.

Finally, there are a number of minor issues with the manuscript that should be fixed prior to publication, such as:

1. In the first paragraph of the introduction, “generate path between high-symmetry points” should probably be “generate paths between high-symmetry points”, i.e. “path” should be “paths”.
2. On page 4/16, just before Equation 2, there appears to be an article missing before “central quantity”, i.e. it should be “as a central quantity” or “as the central quantity”.
3. On page 4/16, in point 6, “Example of highly defected structures” should probably be “Examples of highly defected structures”, i.e. “Example” should be “Examples”.
4. On page 9/16, in the first paragraph of the Methods section, “generated from these supercells removing” should probably be “generated from these supercells by removing”.
5. Finally, it appears that Ref. 12 is now available online at Acta Cryst. A, so should probably be updated to reflect this.

Manuscript: "Insightful classification of crystal structures using deep learning"
Reply to the Reviewers' comments

Reviewers' comments:

Reviewer #2 (Remarks to the Author):

The main concern raised in the previous report was extent to which the machine learning method introduced in this work is dependent on the use of traditional symmetry routines to generate the conventional cell. To address this issue, the authors regenerated the cells using the convention for triclinic symmetry, meaning that the cell generation is independent of its symmetry classification, and also randomized the cell orientations. This approach made little difference to the results (only small quantitative differences in Fig. 4, and some changes to the diffraction pattern for hex and rhl systems in Figs. 2 and 5), successfully demonstrating that their approach does not depend on the routines used to generate the conventional cell.

Other minor suggestions in the previous report included modifying the title and adding some specific example applications to the introduction, both of which have been made appropriately. The authors have also fixed the list of advantages of the method to clarify that the non-uniqueness across space groups is a disadvantage.

Finally, there are a number of minor issues with the manuscript that should be fixed prior to publication, such as:

1. In the first paragraph of the introduction, “generate path between high-symmetry points” should probably be “generate paths between high-symmetry points”, i.e. “path” should be “paths”.
2. On page 4/16, just before Equation 2, there appears to be an article missing before “central quantity”, i.e. it should be “as a central quantity” or “as the central quantity”.
3. On page 4/16, in point 6, “Example of highly defected structures” should probably be “Examples of highly defected structures”, i.e. “Example” should be “Examples”.
4. On page 9/16, in the first paragraph of the Methods section, “generated from these supercells removing” should probably be “generated from these supercells by removing”.
5. Finally, it appears that Ref. 12 is now available online at Acta Cryst. A, so should probably be updated to reflect this.

We thank Ref #2 for pointing this out. The typos have been corrected and they are highlighted in blue in the pdf version of the manuscript. Ref. 12 has also been updated.
